# Change in forced vital capacity and associated subsequent outcomes in patients with newly diagnosed idiopathic pulmonary fibrosis

**DOI:** 10.1186/s12890-015-0161-5

**Published:** 2015-12-29

**Authors:** William M. Reichmann, Yanni F. Yu, Dendy Macaulay, Eric Q. Wu, Steven D. Nathan

**Affiliations:** Analysis Group, Inc., Boston, MA USA; Boehringer Ingelheim Pharmaceuticals, Inc., Ridgefield, CT USA; Analysis Group, Inc., New York, NY USA; Advanced Lung Disease and Transplant Program, Department of Medicine, Inova Fairfax Hospital, Falls Church, VA USA

**Keywords:** Idiopathic pulmonary fibrosis, Forced vital capacity, Healthcare resource utilization, Clinical outcomes

## Abstract

**Background:**

Idiopathic pulmonary fibrosis (IPF) is a rare and serious disease characterized by progressive lung-function loss. Limited evidence has been published on the impact of lung-function loss on subsequent patient outcomes. This study examined change in forced vital capacity (FVC) across IPF patients in the 6 months after diagnosis and its association with clinical and healthcare resource utilization (HRU) outcomes in a real-world setting in the U.S.

**Methods:**

A retrospective chart review was conducted of patients diagnosed with IPF by U.S. pulmonologists. Patient eligibility criteria included: 1) 40 years or older with a confirmed date of first IPF diagnosis with high-resolution computed tomography and/or lung biopsy between 01/2011 and 06/2013; 2) FVC results recorded at first diagnosis (±1 month) and at 6 months (±3 months) following diagnosis. Based on relative change in FVC percent predicted (FVC%), patients were categorized as stable (decline <5 %), marginal decline (decline ≥5 % and <10 %), or significant decline (decline ≥10 %). Physician-reported clinical and HRU outcomes were assessed from ~6 months post-diagnosis until the last contact date with the physician and compared between FVC% change groups. Multivariable Cox proportional-hazards models were constructed to assess risk of mortality, suspected acute exacerbation (AEx), and hospitalization post-FVC% change. Generalized estimating equations were used to account for multiple patients contributed by individual physicians.

**Results:**

The sample included 490 IPF patients contributed by 168 pulmonologists. The mean (SD) age was 61 (11) years, 68 % were male, and the mean (SD) baseline FVC% was 60 % (26 %). 250 (51 %) patients were categorized as stable, 98 (20 %) as marginal decline, and 142 (29 %) as significant decline. The mean (SD) observation time was 583 (287) days. In both unadjusted analysis and multivariable models, significantly worse clinical outcomes and increased HRU were observed with greater lung-function decline.

**Conclusions:**

These findings suggest that nearly half of IPF patients experienced decline in FVC% within ~6 months following IPF diagnosis. Greater FVC% decline was associated with an increased risk of further IPF progression, suspected AEx, mortality, and higher rate of HRU. Management options that slow FVC decline may help improve future health outcomes in IPF.

## Background

Idiopathic pulmonary fibrosis (IPF) is a rare, chronic, and serious pulmonary disease of unknown etiology characterized by the progressive loss of lung function. The prevalence of IPF in the U.S. is estimated to be 14 to 43 per 100,000 person-years and increases with age [1]. More recent incidence and prevalence estimates among the elderly U.S. population are 91 and 495 cases per 100,000 person-years [2]. Following diagnosis, the prognosis is generally poor, with a median survival time of approximately 3–5 years [3, 4]. Patients with IPF may remain stable, progress steadily over time, progress rapidly, or experience episodes of acute deterioration, some of which can be acute exacerbations (AEx) [5]. Until recently, treatment options for IPF patients in the U.S. had been limited to oxygen therapy, pulmonary rehabilitation, and, in select cases, lung transplantation [5, 6]. In October 2014, the Food and Drug Administration approved two new pharmacologic agents for IPF, expanding treatment options [7, 8]. Both drugs have been recommended for use in the 2015 update to the 2011 ATS/ERS/JRS/ALAT IPF clinical practice guidelines [9].

The variable clinical course of IPF presents a challenge to predicting disease progression and managing care of patients [3]. Given the limited treatment options and the substantial impact of the disease on patients’ health outcomes [10, 11], increased evidence regarding the clinical progression of IPF are needed. Previous studies have shown that patients with IPF have significantly higher healthcare resource utilization (HRU) compared to matched controls [12, 13] as well as an increased risk of death [14]. Martinez et al. reported on the clinical course of IPF in a multicenter randomized controlled trial and observed frequent hospitalizations and rapid progression of lung disease in patients who died due to IPF [15]. Further, decline in FVC has been shown to be associated with an increased risk of progression and death [16–18]. However, studies have not examined the relationship between lung-function change and other clinical outcomes, such as AEx or HRU. The research gap arises in part due to difficulty in obtaining measures of lung function at time points throughout the course of disease in the real world. Such data could be useful for key stakeholders, especially with the recent availability of disease modulating therapies for IPF.

This study aimed to address the evidence gap through the use of chart data extracted by treating pulmonologists from a nationwide panel in the U.S. The existing literature have been constrained to either post-hoc analyses of patients from randomized controlled trials or observational studies from single centers, neither of which may be representative of the broader IPF population. The approach used in this study allowed for a study population with a sufficient sample size that would be representative of a variety of treatment settings. The objective of this study was to assess the association of lung-function change with clinical outcomes and IPF-related HRU in patients with newly diagnosed IPF.

## Methods

### Data source

A retrospective chart review was conducted by pulmonologists from a nationally representative panel using an online case report form. The panel comprised over 1,000 pulmonologists working in both academic and non-academic institutions, and covered all regions in the U.S. The pulmonologist panel was demographically similar to those on the American Medical Association master list [19].

### Sample selection

Pulmonologists on the panel were invited via e-mail to participate. Those who accepted the invitation completed a questionnaire to assess their eligibility. Pulmonologists were eligible to participate if they had complete access to their patients’ (inpatient and outpatient) IPF-related medical records. Eligible physicians were then requested to randomly select IPF patients who met the inclusion criteria for this study. IPF patients were eligible for chart abstraction if they met the following criteria: 1) 40 years or older with a confirmed date of first IPF diagnosis with HRCT and/or lung biopsy between January 1, 2011 and June 30, 2013; and 2) the patient’s pulmonary function tests measured using FVC were recorded at or around first IPF diagnosis (±1 month) and at or around 6 months (±3 months) following diagnosis.

Pulmonologists were compensated for their participation and remained anonymous to the study sponsor and vice versa. The electronic case report form and study synopsis were reviewed by the New England Institutional Review Board, which granted exemption from a full review because this non-interventional study collected retrospective, de-identified data. Patient consent forms were not required, and the chart abstraction form did not request any information that could be linked to a patient’s identity, such as name, date of birth, date of death, or social security number.

### Study period

Data were collected from the date of initial IPF diagnosis until last follow-up. The last follow-up date was defined as either: 1) the last contact the pulmonologist had with the patient for those who were alive as of chart abstraction; or 2) the date of death for the deceased. The study period was divided into two time periods, concurrent and subsequent. The “concurrent period” was defined as the time from initial IPF diagnosis date to the FVC measurement closest to 6 months post-IPF diagnosis. The “subsequent period” was defined as the time from the FVC measurement date closest to 6 months post-IPF diagnosis to the last follow-up date (i.e., end of the study period) with no requirement for minimum length of follow-up. The length of the concurrent and subsequent periods therefore varied for patients.

### Independent variables and outcome measures

The main independent variable of interest was relative change in FVC percent predicted (FVC%) over 6 months. The stable group was defined as <5 % decrease in FVC%, the marginal decline group as ≥5 % and <10 % decrease in FVC%, and the significant decline group as ≥10 % decrease in FVC%. Other lung-function measures collected included forced expiratory volume after 1 min (FEV_1_), FEV_1_/FVC ratio, and the single breath diffusing capacity for the lung for carbon monoxide (DL_CO_). The GAP index was also calculated at the initial IPF diagnosis based on the patient’s gender, age, FVC%, and DL_CO_ values [20]. In order to estimate the GAP index for all patients, missing DL_CO_ values were imputed for 108 patients (22 %) using single imputation (see Appendix).

Clinical and IPF-related HRU outcomes were collected during the subsequent period and were based on physician report. Clinical outcomes included all-cause mortality, mortality due to IPF, mortality due to a suspected AEx, suspected AEx, and IPF progression. Suspected AEx was defined by asking participating pulmonologists if a particular outpatient visit, emergency room (ER) visit, or hospitalization was related to an IPF AEx. IPF progression was based on physician assessment. Physicians were asked if the patient had experienced one of the following since the previous visit: progressive dyspnea, increased cough, sustained decrease from baseline in absolute FVC and DL_CO_, progression of fibrosis from baseline on HRCT, AEx, respiratory failure, or new need for supplemental oxygen or increase in oxygen requirements.

IPF-related HRU outcomes were collected in the outpatient, ER, and hospital settings. Outcomes in the outpatient setting included the timing and number of outpatient visits, outpatient visits that were unscheduled and/or for urgent care, and outpatient visits that were for suspected AEx. Also documented were the timing and number of visits where prednisone, azathioprine, and/or N-acetylcysteine were prescribed and where arterial blood gas test, high-resolution computed tomography, chest x-ray, lung biopsy, and/or pulmonary rehabilitation were conducted for IPF. Similar outcomes were documented when patients presented to the ER and hospital settings.

Information on the above clinical and HRU variables was also collected for the concurrent period to assess whether patients in different FVC% change groups had different characteristics in the concurrent period.

### Statistical analysis

Summary statistics for characteristics of the treating physician and their practice were calculated. Patient characteristics at the time of diagnosis were compared between patients in different FVC% change groups.

The incidence rate (IR) or risk of clinical and HRU outcomes was assessed using unadjusted regression analysis with FVC% change group as the only independent variable and was estimated at the per-patient-half-year level for the concurrent period and per-patient-year level for the subsequent period. Negative binomial regression was used to estimate the incidence rate ratio (IRR) and its 95 % confidence interval (CI) for count variables. Logistic regression was used to estimate the odds ratio (OR) and its 95 % CI for binary variables except for mortality. For both models, patient-specific offset terms were used to account for varying follow-up time. For mortality, Kaplan-Meier analysis was performed to obtain the risk of death by 12 months, and an unadjusted Cox proportional hazards regression was used to estimate the hazard ratio (HR) and its 95 % CI. As a sensitivity analysis, the stable and marginal decline groups were combined, and HRU outcomes were compared between this combined group and the significant decline group (See Appendix), i.e., FVC% decline <10 % vs. FVC% decline ≥10 %.

Additionally, multivariable Cox proportional hazards regressions were constructed to assess factors associated with risk of mortality, hospitalization, and suspected AEx. For each model, the main independent variable of interest was the FVC% change group while the control variables consisted of physician’s main practice setting, patient characteristics and symptoms at IPF diagnosis, smoking status, GAP index, suspected AEx in the concurrent period, and use of prednisone and azathioprine in the concurrent period. The combination therapy of prednisone and azathioprine was included per the findings of the PANTHER-IPF clinical trial, which found the triple drug combination of prednisone, azathioprine, and n-acetylcysteine to significantly increase mortality risk relative to placebo [21]. In constructing the Cox regressions, the proportional hazards assumption was tested and found to be satisfied for the FVC% change group variables for all models. The HR and 95 % CI for each risk factor were estimated.

All regression analyses used the generalized estimating equations (GEE) technique to account for correlation from the clustering of multiple patient charts contributed by the same pulmonologist. *P*-values for all pairwise comparisons of FVC% change groups were adjusted for multiple comparisons using a Bonferroni correction, and *p*-values that were less than 0.05 were considered to be statistically significant. All statistical analyses were performed using SAS version 9.3.

## Results

### Physician characteristics

A total of 168 pulmonologists participated in this study, of whom 69 (41.1 %) practiced in an academic setting. The practices were distributed across the Northeast (36.3 %), South (25.0 %), Midwest (20.8 %) and West (17.9 %) of the U.S. The mean (SD) number of years in practice for the pulmonologists was 15.0 (6.4), with each on average contributing data abstracted from 3 patient charts (Appendix: Table 9).

### Patient characteristics

Four-hundred ninety IPF patients were included in this study, of which 250 (51.0 %) were classified as stable, 98 (20.0 %) were classified as having a marginal decline, and 142 (29.0 %) were classified as having a significant decline. The mean (SD) follow-up time for the entire sample was 583 (287) days with the average length of the concurrent and subsequent periods being 176 (56) and 407 (283) days, respectively. The majority of the IPF patients were male (68.4 %), and the average age was 61.1 (10.8) years. The majority of patients were of White race (75.3 %) and were enrolled in commercial/private insurance (48.6 %) or Medicare (43.7 %) (Table 1).

**Table 1:** Baseline characteristics by FVC% change group

	By lung-function change category (based on relative change in FVC%)^a^					
	Stable [A]	Marginal [B]	Significant [C]	*P*-value*		
	(*N* = 250)	(*N* = 98)	(*N* = 142)	[C] vs. [A]	[C] vs. [B]	[B] vs. [A]
Male, N (%)	167 (66.8 %)	70 (71.4 %)	98 (69.0 %)	0.999	0.999	0.999
Age (years), mean (SD)	60.8 (10.8)	62.1 (9.6)	60.9 (11.5)	0.999	0.999	0.930
BMI, mean (SD)	26.7 (4.9)	26.5 (4.4)	26.3 (4.6)	0.999	0.999	0.999
Race, N (%)^b^						
White	183 (73.2 %)	82 (83.7 %)	104 (73.2 %)	0.999	0.193	0.098
Black or African American	34 (13.6 %)	11 (11.2 %)	24 (16.9 %)	0.999	0.650	0.999
Hispanic, Latino, or Spanish Origin	26 (10.4 %)	5 (5.1 %)	13 (9.2 %)	0.999	0.646	0.295
Asian	7 (2.8 %)	1 (1.0 %)	1 (0.7 %)	0.479	0.999	0.952
Other	2 (0.8 %)	0 (0.0 %)	0 (0.0 %)			
Insurance type, N (%)^b^						
Commercial/private insurance	113 (45.2 %)	45 (45.9 %)	80 (56.3 %)	0.193	0.502	0.999
Medicare	109 (43.6 %)	44 (44.9 %)	61 (43.0 %)	0.999	0.999	0.999
Medicaid	26 (10.4 %)	12 (12.2 %)	17 (12.0 %)	0.999	0.999	0.999
Military insurance (VA or active military)	9 (3.6 %)	2 (2.0 %)	3 (2.1 %)	0.999	0.999	0.999
Other	0 (0.0 %)	1 (1.0 %)	0 (0.0 %)	-	-	-
No insurance	8 (3.2 %)	1 (1.0 %)	1 (0.7 %)	0.228	0.999	0.584
Smoking status at diagnosis, N (%)				0.789	0.999	0.999
Never smoked	98 (39.2 %)	44 (44.9 %)	48 (33.8 %)			
Former smoker	124 (49.6 %)	42 (42.9 %)	77 (54.2 %)			
Current smoker	21 (8.4 %)	11 (11.2 %)	16 (11.3 %)			
Unknown/not sure	7 (2.8 %)	1 (1.0 %)	1 (0.7 %)			
Exposure to activities/ environmental agents, N (%)^b^						
Asbestos	10 (4.0 %)	3 (3.1 %)	11 (7.7 %)	0.336	0.650	0.999
Farming/agriculture	27 (10.8 %)	9 (9.2 %)	25 (17.6 %)	0.213	0.131	0.999
Hairdressing	12 (4.8 %)	4 (4.1 %)	4 (2.8 %)	0.999	0.999	0.999
Metal dust	18 (7.2 %)	2 (2.0 %)	10 (7.0 %)	0.999	0.172	0.154
Stone cutting/polishing	7 (2.8 %)	0 (0.0 %)	6 (4.2 %)	0.999	<0.001*	<0.001*
Coal mining	5 (2.0 %)	1 (1.0 %)	2 (1.4 %)	0.999	0.999	0.999
Other	3 (1.2 %)	3 (3.1 %)	2 (1.4 %)	0.999	0.999	0.669
None	182 (72.8 %)	78 (79.6 %)	92 (64.8 %)	0.340	0.037*	0.598
Family history of pulmonary fibrosis, N (%)	16 (6.4 %)	6 (6.1 %)	5 (3.5 %)	0.564	0.923	0.999
Measurement period (days)						
Concurrent, mean (SD)	172.8 (55.5)	174.9 (53.7)	181.6 (56.8)	0.575	0.999	0.999
Subsequent, mean (SD)	438.3 (283.1)	423.0 (280.0)	340.1 (275.9)	0.013*	0.118	0.999
Total follow-up, mean (SD)	611.1 (282.7)	597.9 (285.8)	521.7 (286.6)	0.036*	0.225	0.999

^a^Lung-function change categories were defined as the relative change in FVC% from index to approximately 6 months following IPF diagnosis. "Stable" was defined as decline <5 %. "Marginal" was defined as decline ≥5 % and <10 %, while "Significant" was defined as decline ≥10 %

^b^Physicians were allowed to select multiple values for race, insurance type, and exposure to activities/environmental agents, so counts and percentages may not sum to the total N or 100 %

*All *p*-values were adjusted for multiple comparisons using the Bonferroni correction

At the initial IPF diagnosis date, the mean (SD) FVC of the population was 2.5 (1.0) liters, and the mean (SD) FVC% was 60.4 % (26.1 %), while the mean (SD) DL_CO_ percent predicted was 51.3 % (15.5 %). The mean GAP index was 3.3 (1.5) out of a maximum score of 8 (highest risk). The most common IPF symptoms at diagnosis were dyspnea or shortness of breath (87.6 %), cough (72.7 %), and fatigue or malaise (47.3 %). Gastroesophageal reflux disease was the most commonly reported comorbidity (31.2 %). Generally, patient characteristics were not significantly different among the three FVC% change groups. The significant decline group had lower DL_CO_ and FEV_1_ values than the stable group (47.7 % vs. 53.4 % for DL_CO_, *p* = 0.033; 1.7 vs. 2.0 liters for FEV_1_, *p* = 0.021) (Table 2). The significant decline group also had higher rates of dyspnea and gradual, unintended weight loss than the stable group (93.0 % vs. 83.6 % for dyspnea, *p* = 0.030; 16.9 % vs. 6.8 % for unintended weight loss, *p* = 0.035).

**Table 2:** Clinical characteristics at initial IPF diagnosis by FVC% change group

	By lung-function change category (based on relative change in FVC%)^a^					
	Stable [A]	Marginal [B]	Significant [C]	*P*-value*		
	(*N* = 250)	(*N* = 98)	(*N* = 142)	[C] vs. [A]	[C] vs. [A]	[C] vs. [A]
Comorbidities, N (%)^b^						
Gastroesophageal reflux disease	82 (32.8 %)	26 (26.5 %)	45 (31.7 %)	0.999	0.999	0.999
Cardiac disorder	43 (17.2 %)	28 (28.6 %)	34 (23.9 %)	0.260	0.999	0.053
Metabolic disorder	38 (15.2 %)	20 (20.4 %)	29 (20.4 %)	0.500	0.999	0.642
Other vascular disorder	32 (12.8 %)	23 (23.5 %)	27 (19.0 %)	0.300	0.999	0.069
Pulmonary hypertension	31 (12.4 %)	20 (20.4 %)	16 (11.3 %)	0.999	0.095	0.198
Emphysema	18 (7.2 %)	8 (8.2 %)	15 (10.6 %)	0.783	0.999	0.999
Other pulmonary disorder	14 (5.6 %)	7 (7.1 %)	13 (9.2 %)	0.435	0.999	0.999
Symptoms at IPF diagnosis, N (%)^b^						
Dyspnea/shortness of breath	209 (83.6 %)	88 (89.8 %)	132 (93.0 %)	0.030*	0.999	0.668
Cough	175 (70.0 %)	71 (72.4 %)	110 (77.5 %)	0.428	0.999	0.999
Fatigue or malaise	107 (42.8 %)	47 (48.0 %)	78 (54.9 %)	0.145	0.999	0.999
Rapid, shallow breathing	25 (10.0 %)	11 (11.2 %)	14 (9.9 %)	0.999	0.999	0.999
Gradual, unintended weight loss	17 (6.8 %)	9 (9.2 %)	24 (16.9 %)	0.035*	0.317	0.999
Clubbing	14 (5.6 %)	11 (11.2 %)	17 (12.0 %)	0.309	0.999	0.321
Other	1 (0.4 %)	0 (0.0 %)	2 (1.4 %)	-	-	-
GAP index, mean (SD)^c^	3.3 (1.5)	3.5 (1.5)	3.7 (1.4)	0.149	0.999	0.774
Lung-function measures at IPF diagnosis, mean [median] (SD)						
FVC (liters)	2.6 (1.1)	2.4 (0.9)	2.4 (1.0)	0.821	0.999	0.796
FVC%	61.7 %(26.1 %)	58.5 % (24.0 %)	59.5 % (27.6 %)	0.999	0.999	0.999
FEV_1_ (liters)	2.0 (0.9)	1.8 (0.7)	1.7 (0.6)	0.021*	0.901	0.395
FEV_1_/FVC	79.6 % (12.6 %)	79.9 % (11.8 %)	80.7 % (12.5 %)	0.999	0.999	0.999
DL_CO_ percent predicted	53.4 % (14.4 %)	51.2 % (16.1 %)	47.7 % (16.5 %)	0.033*	0.583	0.962

^a^Lung-function change categories were defined as the relative change in FVC% from index to approximately 6 months following IPF diagnosis. "Stable" was defined as decline <5 %. "Marginal" was defined as decline ≥5 % and <10 %, while "Significant" was defined as decline ≥10 %

^b^Physicians were allowed to select multiple values for comorbidities and symptoms, so counts and percentages may not sum to the total N or 100 %

^c^Missing values for DL_CO_ were imputed using a single imputation method in which observed DL_CO_ was regressed on patient's index FVC%, age, gender, BMI, smoking status at diagnosis, exposure to environmental agents, comorbidities (including cardiac disorder, pulmonary hypertension, emphysema, and gastroesophageal reflux disease), and symptoms at IPF diagnosis (including dyspnea/shortness of breath and gradual, unintended weight loss)

*All *p*-values were adjusted for multiple comparisons using the Bonferroni correction

### Unadjusted analysis of clinical characteristics and HRU in the concurrent period

No significant differences were observed between the FVC% change groups in the concurrent period with regards to rate or risk of suspected AEx and HRU. The exception to this was risk of progression, which was higher for the significant and marginal decline groups compared to the stable group (Table 3).

**Table 3:** Clinical characteristics and healthcare resource utilization in the concurrent period by FVC% change group

	By lung-function change category (based on relative change in FVC%)^a^					
	Stable [A]	Marginal [B]	Significant [C]	Relative effect measure (95 % CI)		
	(*N* = 250)	(*N* = 98)	(*N* = 142)	[C] vs. [A]	[C] vs. [B]	[B] vs. [A]
	6-Month IR per Patient			IRR		
Rate of suspected AEx	0.21	0.25	0.25	1.20 (0.69–2.09)	1.00 (0.55–1.82)	1.20 (0.63–2.28)
	6-Month Risk			OR		
Risk of suspected AEx	14.1 %	16.7 %	20.4 %	1.56 (0.78–3.15)	1.27 (0.56–2.91)	1.23 (0.56–2.70)
Risk of progression	23.4 %	38.7 %	48.6 %	3.09 (1.67–5.72)	1.50 (0.75–3.00)	2.07 (1.07–3.97)
	6-Month IR per Patient			IRR		
Rate of IPF-related outpatient visits	1.85	1.95	1.78	0.96 (0.82–1.12)	0.91 (0.75–1.12)	1.05 (0.87–1.27)
Unscheduled and/or urgent care	0.16	0.23	0.22	1.42 (0.64–3.15)	0.97 (0.48–1.96)	1.46 (0.76–2.80)
For a suspected AEx	0.17	0.19	0.19	1.16 (0.58–2.31)	1.04 (0.52–2.09)	1.11 (0.54–2.29)
Rate of IPF-related ER visits	0.18	0.16	0.14	0.81 (0.31–2.11)	0.87 (0.37–2.01)	0.94 (0.40–2.17)
For a suspected AEx	0.04	0.02	0.05	1.15 (0.34–3.86)	2.46 (0.41–14.72)	0.47 (0.07–3.17)
Rate of IPF-related hospitalizations	0.06	0.08	0.05	0.80 (0.20–3.12)	0.59 (0.17–1.98)	1.36 (0.35–5.20)
For a suspected AEx	0.04	0.06	0.04	0.96 (0.19–4.82)	0.63 (0.17–2.32)	1.52 (0.27–8.60)
Rate of hospital days	0.39	0.48	0.25	0.63 (0.10–3.82)	0.52 (0.10–2.62)	1.22 (0.28–5.26)
Rate of ICU stays	0.03	0.03	0.02	0.59 (0.07–4.97)	0.47 (0.07–2.95)	1.25 (0.21–7.58)
Rate of ICU days	0.08	0.18	0.07	0.79 (0.05–12.62)	0.36 (0.03–4.05)	2.16 (0.23–20.59)

*AEx* Acute exacerbation*, IR* incidence rate*, IRR* incidence rate ratio*, OR odds ratio*

^a^Lung-function change categories were defined as the relative change in FVC% from index to approximately 6 months following IPF diagnosis. "Stable" was defined as decline <5 %. "Marginal" was defined as decline ≥5 % and <10 %, while "Significant" was defined as decline ≥10 %

### Unadjusted analysis of clinical outcomes in the subsequent period

Significantly higher risk of worse clinical outcomes were observed in groups with greater FVC% decline, including risk of progression (significant vs. stable, >3-fold higher), risk of suspected AEx (significant vs. stable, >4-fold higher; marginal vs. stable, >2-fold higher), all-cause mortality (significant vs. stable, >5-fold higher; significant vs. marginal, 1.8-fold higher; marginal vs. stable, >2-fold higher), mortality due to IPF (significant vs. stable, >6-fold higher; significant vs. marginal, 1.9-fold higher; marginal vs. stable, >3-fold higher), and mortality due to a suspected AEx (significant vs. stable,>3-fold higher; marginal vs. stable, >3-fold higher) (Table 4). In the stable and marginal decline groups, mortality due to a suspected AEx was similar to all-cause and IPF-related mortality, indicating that suspected AEx was likely the main driver of mortality in these groups. However, in the significant decline group, mortality due to suspected AEx was approximately half that of all-cause and IPF-related mortality, indicating that progression of IPF might be the main driver of mortality in this group.

**Table 4:** Clinical outcomes in the subsequent period by FVC% change group

	By lung-function change category (based on relative change in FVC%)^a^					
	Stable [A]	Marginal [B]	Significant [C]	Relative effect measure (95 % CI)		
	(*N* = 250)	(*N* = 98)	(*N* = 142)	[C] vs. [A]	[C] vs. [B]	[B] vs. [A]
	12-Month IR per Patient			IRR		
Rate of suspected AEx	0.26	0.47	0.74	2.87 (1.71–4.82)	1.58 (0.95–2.64)	1.82 (1.02–3.23)
	12-Month Risk			OR		
Risk of suspected AEx	19.2 %	37.1 %	50.9 %	4.37 (2.09–9.16)	1.76 (0.81–3.80)	2.49 (1.28–4.82)
Risk of progression	62.6 %	76.2 %	85.6 %	3.56 (1.66–7.64)	1.86 (0.81–4.26)	1.92 (0.91–4.04)
Mortality^b^	Risk by 12 Months			HR		
Death due to any cause	6.4 %	13.1 %	28.0 %	5.05 (2.75–9.27)	1.85 (1.05–3.26)	2.73 (1.51–4.94)
Death due to IPF	5.5 %	10.3 %	24.3 %	6.23 (2.96–13.14)	1.94 (1.01–3.70)	3.22 (1.59–6.53)
Death due to AEx	5.0 %	9.3 %	13.1 %	3.91 (1.56–9.84)	1.22 (0.54–2.77)	3.21 (1.41–7.30)

*AEx* acute exacerbation*, IR* incidence rate*, IRR incidence rate ratio, OR* odds ratio, *HR hazard ratio*

^a^Lung-function change categories were defined as the relative change in FVC% from index to approximately 6 months following IPF diagnosis. "Stable" was defined as decline <5 %. "Marginal" was defined as decline ≥5 % and <10 %, while "Significant" was defined as decline ≥10 %

^b^Kaplan-Meier survival analysis was used to estimate the risk of death by 12 months

### Unadjusted analysis of HRU outcomes in the subsequent period

During the subsequent period, the overall IRs of outpatient visits, ER visits, and hospitalization were 2.39, 0.43, and 0.34 per patient-year, respectively. The corresponding overall 12-month risks of outpatient visits, ER visits, and hospitalization were 96.6 %, 21.5 % and 15.2 %, respectively. Significantly higher rates were observed in groups with greater FVC% decline for the following variables: outpatient visits for a suspected AEx, unscheduled and/or urgent care outpatient visits, hospitalization for a suspected AEx, treatments and medications prescribed at outpatient visits, arterial blood gas tests used in the ER setting, number of days in the hospital, ICU stays, and tests used in the hospital setting (Table 5). Similar results were found when the marginal and stable groups were combined and compared with the significant group (Appendix: Table 10).

**Table 5:** Healthcare resource utilization outcomes in the subsequent period by FVC% change group

By lung-function decline category (based on relative change in FVC%)^a^						
	Stable [A]	Marginal [B]	Significant [C]	Relative effect measure (95 % CI)		
	(*N* = 250)	(*N* = 98)	(*N* = 142)	[C] vs. [A]	[C] vs. [B]	[B] vs. [A]
	12-Month IR per Patient			IRR		
Rate of IPF-related outpatient visits	2.24	2.57	2.55	1.14 (0.89–1.46)	0.99 (0.76–1.30)	1.15 (0.91–1.45)
Unscheduled and/or urgent care	0.18	0.38	0.54	3.01 (1.51–5.97)	1.41 (0.78–2.55)	2.13 (1.05–4.34)
For a suspected AEx	0.19	0.33	0.54	2.81 (1.47–5.40)	1.64 (0.86–3.11)	1.72 (0.80–3.69)
Rate of outpatient visits indicating that the following medications/treatments were prescribed for IPF						
Prednisone	1.06	1.16	1.60	1.51 (1.08–2.10)	1.38 (1.02–1.86)	1.10 (0.79–1.52)
Azathioprine	0.36	0.31	0.54	1.51 (0.78–2.93)	1.72 (0.84–3.53)	0.88 (0.44–1.76)
N-acetylcysteine	0.65	0.70	0.79	1.22 (0.79–1.89)	1.13 (0.67–1.90)	1.08 (0.69–1.70)
Rate of outpatient visits that included the following tests/procedures						
Arterial blood gas test	0.17	0.23	0.34	2.04 (0.77–5.38)	1.46 (0.53–4.03)	1.40 (0.55–3.56)
HRCT	0.23	0.35	0.35	1.55 (0.74–3.21)	1.02 (0.45–2.31)	1.51 (0.76–2.99)
Chest X-ray	0.48	0.66	0.60	1.23 (0.75–2.02)	0.90 (0.55–1.48)	1.36 (0.84–2.20)
Lung biopsy	0.03	0.04	0.01	0.44 (0.02–8.70)	0.31 (0.02–4.91)	1.42 (0.13–15.70)
Pulmonary rehabilitation	0.18	0.19	0.26	1.44 (0.79–2.63)	1.35 (0.58–3.11)	1.07 (0.52–2.23)
Rate of IFP-related ER visits	0.35	0.54	0.53	1.53 (0.71–3.28)	0.98 (0.51–1.87)	1.56 (0.66–3.71)
For a suspected AEx	0.13	0.22	0.29	2.22 (0.95–5.21)	1.32 (0.60–2.87)	1.69 (0.67–4.27)
Rate of ER visits that included the following tests/procedures						
Arterial blood gas test	0.15	0.35	0.33	2.24 (1.01–4.99)	0.95 (0.45–2.00)	2.36 (1.01–5.49)
HRCT	0.09	0.18	0.20	2.17 (0.85–5.58)	1.09 (0.37–3.22)	1.99 (0.60–6.67)
Chest X-ray	0.20	0.28	0.30	1.51 (0.69–3.32)	1.10 (0.55–2.21)	1.37 (0.61–3.09)
Supplemental oxygen therapy	0.17	0.23	0.29	1.74 (0.73–4.14)	1.26 (0.52–3.04)	1.39 (0.46–4.20)
Rate of IPF-related hospitalizations	0.24	0.32	0.61	2.53 (0.70–9.10)	1.89 (0.94–3.78)	1.34 (0.34–5.29)
For a suspected AEx	0.09	0.21	0.43	4.69 (1.84–11.99)	2.05 (1.03–4.08)	2.29 (0.82–6.35)
Rate of hospital days	1.13	2.32	5.25	4.63 (1.31–16.42)	2.27 (0.86–5.97)	2.05 (0.66–6.33)
Rate of ICU stays	0.05	0.17	0.26	4.95 (1.20–20.41)	1.53 (0.59–3.99)	3.23 (0.72–14.53)
Rate of ICU days	0.30	0.69	1.22	4.05 (0.79–20.91)	1.75 (0.58–5.35)	2.31 (0.54–9.83)
Rate of hospitalizations that included the following tests/procedures						
Arterial blood gas test	0.10	0.19	0.35	3.58 (1.33–9.64)	1.87 (0.73–4.80)	1.92 (0.61–6.00)
Mechanical ventilation	0.04	0.11	0.14	3.29 (0.63–17.23)	1.33 (0.38–4.70)	2.47 (0.57–10.73)
Non-invasive ventilation	0.02	0.08	0.22	10.17 (3.25–31.86)	2.80 (1.05–7.51)	3.63 (0.90–14.70)
Supplemental oxygen therapy	0.07	0.13	0.28	3.95 (1.38–11.29)	2.11 (0.93–4.82)	1.87 (0.62–5.66)
	12-Month Risk			OR		
Risk of having ≥1 outpatient visit	97.5 %	97.3 %	95.0 %	0.48 (0.14–1.62)	0.52 (0.12–2.20)	0.93 (0.23–3.76)
Unscheduled or urgent care	11.4 %	26.9 %	34.5 %	4.10 (1.95–8.63)	1.43 (0.69–2.98)	2.87 (1.39–5.92)
For a suspected AEx	12.7 %	21.9 %	33.8 %	3.52 (1.62–7.67)	1.82 (0.82–4.05)	1.93 (0.90–4.15)
Risk of having ≥1 ER visit	14.4 %	29.4 %	32.4 %	2.85 (1.36–5.98)	1.15 (0.53–2.52)	2.47 (1.22–5.03)
For a suspected AEx	7.8 %	16.7 %	22.1 %	3.33 (1.40–7.94)	1.42 (0.57–3.54)	2.35 (0.98–5.65)
Risk of having ≥1 hospitalizations	7.8 %	19.1 %	30.0 %	5.04 (2.13–11.93)	1.82 (0.71–4.66)	2.76 (1.14–6.70)
Intensive care unit (ICU) stay	2.4 %	8.4 %	12.4 %	5.86 (1.43–24.01)	1.56 (0.41–5.90)	3.77 (0.88–16.03)
For a suspected AEx	5.9 %	15.1 %	27.8 %	6.08 (2.45–15.07)	2.16 (0.84–5.54)	2.81 (1.10–7.20)

*IR* incidence rate*, IRR* incidence rate ratio*, OR* odds ratio

^a^Lung-function change categories were defined as the relative change in FVC% from index to approximately 6 months following IPF diagnosis. "Stable" was defined as decline <5 %. "Marginal" was defined as decline ≥5 % and <10 %, while "Significant" was defined as decline ≥10 %

### Multivariable analysis

Results of the Cox proportional hazards regressions showed that greater FVC% decline was associated with a higher risk of mortality, hospitalization, and suspected AEx in the subsequent period, adjusting for demographic and clinical characteristics. In addition, suspected AEx in the concurrent period was found to be associated with higher risk of mortality (HR = 2.59, *p* < 0.001), hospitalization (HR = 1.86, *p* = 0.030), and subsequent suspected AEx (HR = 2.98, *p* < 0.001) in the subsequent period (Tables 6, 7, and 8).

**Table 6:** Multivariable Cox proportional hazards regression on mortality in the subsequent period

	Mortality^a^	
	Hazard ratio (95 % CI)	*P*-value*
Race		
White vs. non-white	0.70 (0.39–1.26)	0.231
Lung-function decline group		
Marginal decline vs. stable	2.38 (1.04–5.45)	0.036*
Significant decline vs. stable	4.42 (2.01–9.71)	<0.001*
BMI		
25–30 vs. < 25	0.53 (0.29–0.97)	0.038*
≥30 vs. < 25	0.38 (0.18–0.80)	0.012*
Comorbidities		
Cardiac disorder vs. no cardiac disorder	1.63 (0.94–2.82)	0.080
Pulmonary hypertension vs. no pulmonary hypertension	2.53 (1.33–4.80)	0.005*
Emphysema vs. no emphysema	1.49 (0.67–3.35)	0.328
Gastroesophageal reflux disease vs. no gastroesophageal reflux disease	1.10 (0.64–1.92)	0.725
Smoking status		
History of smoking vs. no history of smoking	1.08 (0.64–1.83)	0.773
Suspected AEx in the concurrent period		
Yes vs. no	2.59 (1.49–4.53)	<0.001*
Use of prednisone and azathioprine in the concurrent period		
Both vs. neither	2.48 (1.19–5.19)	0.016*
Prednisone only vs. neither	1.41 (0.66–3.01)	0.376
Azathioprine only vs. neither	1.11 (0.18–7.01)	0.909
Symptoms at initial IPF diagnosis		
Dyspnea vs. no dyspnea	1.29 (0.48–3.48)	0.609
Weight loss vs. no weight loss	1.95 (1.02–3.73)	0.044*
Physician's main practice setting		
Academic vs. non-academic	0.95 (0.51–1.77)	0.876
GAP index (per unit increase)^b^	1.19 (0.98–1.44)	0.080

^a^Multivariable Cox proportional hazard regression was used to estimate the hazard ratio, 95 % confidence interval and p-value, accounting for physician clustering using generalized estimating equations

^b^The hazard ratio for the GAP index was estimated for every one point increase in GAP index

*P-values less than 0.05 are indicated with an asterisk (*)

**Table 7:** Multivariable Cox proportional hazards regression on hospitalization in the subsequent period

	Hospitalization^a^	
	Hazard Ratio (95 % CI)	*P*-value*
Race		
White vs. non-white	0.50 (0.32–0.79)	0.003*
Lung-function decline group		
Marginal decline vs. stable	2.50 (1.06–5.91)	0.033*
Significant decline vs. stable	3.37 (1.62–7.00)	<0.001*
BMI		
25–30 vs. <25	0.59 (0.34–1.03)	0.061
≥30 vs. <25	0.95 (0.51–1.74)	0.861
Comorbidities		
Cardiac disorder vs. no cardiac disorder	1.87 (1.06–3.32)	0.032*
Pulmonary hypertension vs. no pulmonary hypertension	2.09 (1.15–3.83)	0.017*
Emphysema vs. no emphysema	1.44 (0.62–3.36)	0.399
Gastroesophageal reflux disease vs. no gastroesophageal reflux disease	1.08 (0.67–1.73)	0.746
Smoking status		
History of smoking vs. no history of smoking	1.02 (0.61–1.70)	0.935
Suspected AEx in the concurrent period		
Yes vs. no	1.86 (1.06–3.26)	0.030*
Use of prednisone and azathioprine in the concurrent period		
Both vs. neither	1.24 (0.59–2.62)	0.575
Prednisone only vs. neither	1.25 (0.72–2.16)	0.428
Azathioprine only vs. neither	0.74 (0.09–6.38)	0.780
Symptoms at initial IPF diagnosis		
Dyspnea vs. no dyspnea	1.43 (0.43–4.80)	0.562
Weight loss vs. no weight loss	1.45 (0.77–2.76)	0.250
Physician's main practice setting		
Academic vs. non-academic	0.89 (0.51–1.55)	0.680
GAP index (per unit increase)^b^	1.23 (1.04–1.46)	0.018*

^a^Multivariable Cox proportional hazard regression was used to estimate the hazard ratio, 95 % confidence interval and p-value, accounting for physician clustering using generalized estimating equations

^b^The hazard ratio for the GAP index was estimated for every one point increase in GAP index

*P-values less than 0.05 are indicated with an asterisk (*)

**Table 8:** Multivariable Cox proportional hazards regression on suspected acute exacerbation in the subsequent period

	Suspected Acute Exacerbation^a^	
	Hazard Ratio (95 % CI)	*P*-value*
Race		
White vs. non-white	0.63 (0.45–0.88)	0.007*
Lung-function decline group		
Marginal decline vs. stable	2.02 (1.13–3.59)	0.011*
Significant decline vs. stable	2.86 (1.69–4.85)	<0.001*
BMI		
25–30 vs. <25	0.82 (0.53–1.26)	0.362
≥30 vs. <25	0.75 (0.45–1.23)	0.245
Comorbidities		
Cardiac disorder vs. no cardiac disorder	1.57 (1.04–2.37)	0.031*
Pulmonary hypertension vs. no pulmonary hypertension	1.65 (1.03–2.63)	0.036*
Emphysema vs. no emphysema	1.89 (1.02–3.50)	0.043*
Gastroesophageal reflux disease vs. no gastroesophageal reflux disease	1.07 (0.74–1.54)	0.736
Smoking status		
History of smoking vs. no history of smoking	0.91 (0.64–1.31)	0.608
Suspected AEx in the concurrent period		
Yes vs. no	2.98 (1.92–4.62)	<0.001*
Use of prednisone and azathioprine in the concurrent period		
Both vs. neither	1.40 (0.76–2.55)	0.276
Prednisone only vs. neither	1.23 (0.81–1.87)	0.335
Azathioprine only vs. neither	0.99 (0.32–3.05)	0.980
Symptoms at initial IPF diagnosis		
Dyspnea vs. no dyspnea	1.39 (0.68–2.83)	0.368
Weight loss vs. no weight loss	1.16 (0.66–2.05)	0.595
Physician's main practice setting		
Academic vs. non-academic	1.02 (0.69–1.51)	0.910
GAP index (per unit increase)^b^	1.07 (0.94–1.22)	0.301

^a^Multivariable Cox proportional hazard regression was used to estimate the hazard ratio, 95 % confidence interval and p-value, accounting for physician clustering using generalized estimating equations

^b^The hazard ratio for the GAP index was estimated for every one point increase in GAP index

*P-values less than 0.05 are indicated with an asterisk (*)

## Discussion

This study was a retrospective chart review of patients diagnosed with IPF designed to investigate the relationship of FVC% change with clinical and HRU outcomes. Overall, clinical characteristics and HRU were similar between FVC% change groups at initial IPF diagnosis and in the concurrent period. However, both unadjusted analysis and multivariable analysis showed that greater FVC% decline was associated with worse clinical outcomes and increased HRU during the subsequent period. In addition, experiencing a suspected AEx in the concurrent period was found to be a significant risk factor for mortality, hospitalization, and subsequent suspected AEx.

To our knowledge, this is one of the largest chart review studies conducted for IPF patients in the U.S. and the only one that attempts to link FVC% change with HRU outcomes [16, 18, 22]. Results for clinical outcomes were similar to results from observational studies and randomized clinical trials [14, 16, 17, 23–26]. In particular, recent studies by Zappala et al. and Salisbury et al. have reported that patients with significant or marginal FVC declines were at higher risk of death compared to those with stable disease or not significant FVC decline [16, 26]. Results from the current study were reasonably concordant, especially for the comparison of the marginal FVC% decline and stable groups. Results for AEx were also in line with prior studies. For instance, Kondoh et al. found a higher risk of subsequent AEx for patients with a significant FVC decline (i.e., ≥10 %) at 6 months compared to those without a significant decline, reporting a HR of 2.6 (*p* = 0.049) [27]. The HR for our significant decline group relative to the stable group was similar at 2.86.

Beyond comparable findings, our study adds to the IPF literature by assessing the association of FVC% change with HRU with a large cohort of IPF patients from a variety of practice settings. One recently published study reported that the proportion of hospitalization was 25 % among patients with IPF and that 37 % of hospitalizations resulted in an ICU stay [28]; our study reported 12-month risks of 15 % and 39 % for these outcomes, respectively, and additionally links them to FVC% decline. Comparison of our results to a claims study published in 2012 demonstrates that annual rates of outpatient visits and ER visits are similar, and the annual rate of hospitalization in our study is slightly lower (0.53 vs. 0.34) than the claims study [12]. Our lower rate of hospitalization is likely attributed to the fact that 1) the claims study reported all-cause hospitalizations, while our study focused on IPF-related hospitalizations; 2) we required patients to have a second FVC measurement approximately 6 months after diagnosis. Requiring a second FVC measurement may have excluded the most severe of patients who may have been hospitalized and died early in their disease course.

In addition to evaluating the relationship between FVC% change and outcomes, our multivariable models provide information on patient characteristics associated with mortality, hospitalization, and AEx, adding to the literature on risk factors for these outcomes. AEx in the concurrent period was a consistent and strong risk factor across all these outcomes and was the strongest predictor of a subsequent AEx. Combination therapy with prednisone and azathioprine was associated with a significantly higher mortality risk relative to use of neither drug with an HR of 2.48 (*p* = 0.016), which is consistent with the findings of the PANTHER-IPF trial [21].

Select comorbidities were also significant predictors of worse outcomes as determined through our multivariable Cox regression models. In particular, pulmonary hypertension was a strong risk factor of all three adverse outcomes. While our result for mortality is concordant with the findings of numerous other studies, there is only a single prior report attesting to a relationship between pulmonary hypertension and AEx [29–32]. Furthermore, our finding of non-Caucasian patients being associated with worse outcomes adds to the literature on the disparities in health outcomes among IPF patients.

### Strengths and limitations

Our study has several strengths. The study population came from different treating settings, including patients treated in the community, which is likely more reflective of real-world practice in IPF. A large number of patient charts were contributed by pulmonologists from a national physician panel, allowing for a broad sample of IPF patients across the U.S. Patient charts provided more comprehensive individual patient details and clinical granularity, which are not commonly collected in administrative claims data. Thus, our data source helps to better understand and capture underlying risk factors and allowed us to adjust for important observed confounders in the multivariable regression models. IPF diagnosis was confirmed by HRCT or biopsy as reported by the pulmonologist and is likely to be more accurate than a claims-based IPF patient identification algorithm.

This study is subject to the limitations of retrospective studies. To account for temporal trends in IPF care and management, we sampled patients who were recently diagnosed with IPF. Consequently, the findings may not be applicable to patients diagnosed with IPF prior to 2011. Also, there is the potential for incomplete patient chart information and data entry errors from participating pulmonologists. To address these possible limitations, patient inclusion criteria required pulmonologist access to the patient’s complete inpatient and outpatient medical records related to IPF post-diagnosis. Real-time data checks for consistency and logic of responses were included in the online case report form to reduce the likelihood of data entry errors, such as ensuring that the dates of specific IPF-related events collected in the follow-up period were after the initial IPF diagnosis date.

Our study did not further stratify patients who had smaller declines or improvement in FVC% within the stable group. Even though this group was classified as being stable, patients in this group had reasonably high risk of progression (62.6 %), suspected AEx (19.2 %), and all-cause mortality (6.4 %) over 12 months (Table 4). They also utilized a significant amount of resources in the subsequent period. Future studies teasing out the impact of smaller declines in FVC%, perhaps in conjunction with other measures of progression, may provide insight.

The pulmonologist-reported suspected AEx in this study is another limitation, because the determination likely does not reflect the definition established by Collard et al. in 2007 [3]. More specifically, pulmonologists may use less stringent criteria in their practice settings when classifying AEx due to their patients’ inability to undergo certain procedures required by the definition. Consequently, the rate of suspected AEx observed in this study may be higher than the rate of AEx reported in clinical trials [5, 33]. We speculate that some cases of increased respiratory compromise for other reasons may have been erroneously labeled as a suspected AEx, a term that has indeed been recently defined in the literature [34]. On the other hand, our study provides insight into how AEx are perceived and defined in real-world clinical practice. As demonstrated in our study, these events still have profound implications on downstream HRU even if based on less strict criteria.

Lastly, our study might not have included patients on both ends of the disease spectrum. Specifically, requiring that eligible patients have two FVC measures may have excluded patients with more severe disease who were unable to perform lung-function tests or those who did not survive long enough to receive the subsequent FVC assessment. On the other end of the spectrum, there might have been some milder, more stable patients who were not seen or did not have lung-function testing within the timeline specified in this study.

## Conclusions

In summary, our study demonstrates that greater FVC% decline in the first 6 months after the initial IPF diagnosis is associated with worse clinical outcome and increased IPF-related HRU. The incremental burden of FVC% decline on patients and the healthcare system may underscore the importance of preservation of lung function in IPF patients. Future studies examining treatments that help slow lung function deterioration are warranted along with additional studies identifying predictors of patients at greatest risk of FVC% decline in the months following diagnosis and evaluating variation in effect of lung-function change across subgroups of patients. Our study further highlights the need for definitions and categorization of IPF worsening events that are more applicable to IPF physicians’ daily practice and capture less severe events than those defined by the Collard definition. Finally, management options that ameliorate declines in FVC may have an impact on and help to improve health outcomes in patients with IPF.

## Appendix

**Table 9:** Physician characteristics

	Physicians (*N* = 168)		
	N (%) or Mean (SD)	Median	[Range]
Practice characteristics			
Main practice setting, N (%)			
Academic	69 (41.1 %)		
Non-academic	99 (58.9 %)		
Practice location, N (%)			
Northeast	61 (36.3 %)		
South	42 (25.0 %)		
Midwest	35 (20.8 %)		
West	30 (17.9 %)		
Physician characteristics			
Years in pulmonology practice, mean (SD) median [min, max]	15.0 (6.4)	14	[3, 30]
Percentage of time spent in inpatient vs. outpatient settings, mean (SD) median [min, max]			
Inpatient settings	39.0 (17.2)	40	[5, 90]
Outpatient settings	61.0 (17.2)	60	[10, 95]
Participation in IPF-related research during the past year, N (%)^a,b^			
IPF clinical trial involvement	20 (11.9 %)		
Other IPF research involvement	13 (7.7 %)		
None	138 (82.1 %)		
Physician-reported profile of treated patients (January 1, 2011 – June 20, 2013)			
Number of patients diagnosed with interstitial lung disease (ILD), mean (SD) median [min, max]	132.7 (182.4)	60	[4, 1000]
Number of ILD patients diagnosed with IPF	76.2 (93.5)	45	[3, 600]
Number of deaths within 6 months of IPF diagnosis	10.0 (17.4)	5	[0, 150]
Number of deaths within 12 months of IPF diagnosis	20.6 (38.7)	8	[0, 350]
Percent of ILD patients diagnosed with IPF	68.4 (26.3)	72	[8, 100]
Percent of IPF patients dying within 6 months of IPF diagnosis	12.3 (11.4)	10	[0, 60]
Percent of IPF patients dying within 12 months of IPF diagnosis	26.0 (22.6)	20	[0, 94]
Number of charts abstracted^c^, mean (SD) median [min, max]	2.9 (1.2)	3	[1, 4]

^a^Research involvement includes IPF-related basic clinical research, epidemiology studies, outcomes studies, etc.

^b^Physicians were allowed to select multiple values for type of research involvement, so counts and percentages may not sum to the total N or 100 %

^c^Physicians have been permitted to abstract a maximum of 4 patient charts

### Explanation of imputation for missing value in DL_CO_

Missing values for DL_CO_ were imputed using a single imputation method based on a linear regression in which observed DL_CO_ was regressed on patient's index FVC%, age, gender, BMI, smoking status at diagnosis, exposure to environmental agents, comorbidities (including cardiac disorder, pulmonary hypertension, emphysema, and gastroesophageal reflux disease), and symptoms at IPF diagnosis (including dyspnea/shortness of breath and gradual, unintended weight loss).

**Table 10:** Healthcare resource utilization outcomes in the subsequent period

	By lung-function change category (based on relative change in FVC%)^a^			
	All Patients	Stable or Marginal (Δ < 10 %) [A]	Significant (Δ ≥ 10 %) [B]	Relative Effect Measure (95 % CI)
	(*N* = 490)	(*N* = 348)	(*N* = 142)	[B] vs. [A]
Subsequent Period				
	12-Month IR per Patient			IRR
Rate of IPF-related outpatient visits	2.39	2.34	2.56	1.09 (0.91–1.32)
Unscheduled and/or urgent care	0.30	0.24	0.55	2.25 (1.45–3.50)
For a suspected AEx	0.30	0.24	0.54	2.28 (1.47–3.51)
Rate of outpatient visits indicating that the following medications/treatments were prescribed for IPF				
Prednisone	1.22	1.09	1.60	1.47 (1.16–1.85)
Azathioprine	0.39	0.34	0.54	1.57 (0.96–2.57)
N-acetylcysteine	0.70	0.67	0.79	1.19 (0.85–1.67)
Rate of outpatient visits that included the following tests/procedures				
Arterial blood gas test	0.25	0.19	0.35	1.80 (0.90–3.60)
High-resolution computed tomography	0.29	0.27	0.36	1.32 (0.75–2.33)
Chest X-ray	0.55	0.54	0.60	1.11 (0.77–1.59)
Lung biopsy	0.03	0.03	0.01	0.39 (0.05–3.44)
Pulmonary rehabilitation	0.21	0.19	0.26	1.40 (0.86–2.27)
Rate of IPF-related ER visits	0.43	0.41	0.53	1.30 (0.80–2.12)
For a suspected AEx	0.19	0.16	0.29	1.84 (1.04–3.27)
Rate of ER visits that included the following tests/procedures				
Arterial blood gas test	0.24	0.21	0.34	1.63 (0.95–2.81)
High-resolution computed tomography	0.14	0.12	0.20	1.71 (0.88–3.30)
Chest X-ray	0.24	0.22	0.30	1.36 (0.79–2.32)
Supplemental oxygen therapy	0.21	0.18	0.29	1.58 (0.89–2.79)
Rate of IFP-related hospitalizations	0.34	0.26	0.61	2.31 (1.10–4.85)
For a suspected AEx	0.20	0.13	0.43	3.46 (2.00–5.98)
Rate of hospital days not for a suspected AEx	0.53	0.44	0.81	1.84 (0.38–9.07)
Rate of hospital days for a suspected AEx	2.06	1.07	4.50	4.19 (1.69–10.41)
Rate of ICU stays	0.13	0.09	0.28	3.07 (1.38–6.83)
Rate of ICU days	0.62	0.42	1.22	2.92 (1.12–7.63)
Rate of hospitalizations that included the following tests/procedures				
Arterial blood gas test	0.18	0.12	0.36	2.87 (1.48–5.54)
Mechanical ventilation	0.08	0.07	0.16	2.47 (0.85–7.12)
Non-invasive ventilation	0.08	0.04	0.20	5.50 (2.90–10.45)
Supplemental oxygen therapy	0.13	0.09	0.28	3.22 (1.69–6.17)

*IR* Incidence rate, *IRR* Incidence rate ratio

^a^Lung-function change categories were defined as the relative change in FVC% from index to approximately 6 months following IPF diagnosis. "Stable or Marginal" was defined as decline less than 10 %, while "Significant" was defined as decline greater than or equal to 10 %

## Abbreviations

AEx: Acute exacerbation
ALAT: Latin American Thoracic Association
ATS: American Thoracic Society
BMI: Body mass index
CI: Confidence interval
DL_CO_: Single breath diffusing capacity for the lung for carbon monoxide
ER: Emergency room
ERS: European Respiratory Society
FEV_1_: Forced expiratory volume after 1 min
FVC: Forced vital capacity
FVC%: FVC percent predicted
GAP index: Gender-age-physiology index
GEE: Generalized estimating equations
HR: Hazard ratio
HRCT: High-resolution computed tomography
HRU: Healthcare resource utilization
ICU: Intensive care unit
IPF: Idiopathic pulmonary fibrosis
IR: Incidence rate
IRR: Incidence rate ratio
JRS: Japanese Respiratory Society
OR: Odds ratio
SD: Standard deviation
